# 59821 Brain Mapping Addiction

**DOI:** 10.1017/cts.2021.443

**Published:** 2021-03-30

**Authors:** Brianna Brie Evans, Sarah Ballard, Kyra Newmaster, Yongsoo Kim, Sue Grigson

**Affiliations:** Pennsylvania State College of Medicine, Neural and Behavioral Sciences Department

## Abstract

ABSTRACT IMPACT: Gaining a better understanding on the role of opioids in opioid use disorder (OUD) can help us find better diagnostics, treatments, and procedures to treat the disorder. OBJECTIVES/GOALS: While we are familiar with brain areas and pathways that are implicated in opioid use disorder (OUD), we do not have a full understanding of the neural circuits activated upon drug exposure. METHODS/STUDY POPULATION: In order to identify areas of the brain most activated by opioids, we ran a pilot study using transgenic cFos-GFP mice that were injected with saline or heroin and examined the brain-wide activity patterns using a quantitative high-resolution mapping method. We observed many brain regions highly activated upon drug exposure. To examine cFos based brain activation in rats, we also ran a pilot study using a tissue clearing and 3D immunolabeling method combined with light sheet microscopy. RESULTS/ANTICIPATED RESULTS: We would expect to see higher cFos activation for brain areas in the reward pathway [including the Nucleus Accumbens (NAc), Ventral Tegmental Area (VTA), Prefrontal Cortex (PFC)] in heroin animals compared to saline animals. We can also expect higher activation in more novel areas like the lateral hypothalamus. DISCUSSION/SIGNIFICANCE OF FINDINGS: If we are able to track OUD effects through imaging in mice and rats, this can help us find better diagnostics, therapeutics, and procedures to treat the disorder. We can also eventually have a human brain atlas that outlines these affected areas as well in order to gain a better understanding on OUD particularly in the human population.

